# Plasma H3Cit-DNA Discriminates Between Cancer and Inflammation in a Cohort of Patients with Unspecific Cancer Symptoms

**DOI:** 10.1007/s10753-024-02085-4

**Published:** 2024-06-28

**Authors:** Fredrika Wannberg, Viktoria Hjalmar, Henry Ng, Caroline Johansson, Fay Probert, Mia Phillipson, Mikael Åberg, Max Gordon, Nigel Mackman, Axel Rosell, Charlotte Thålin

**Affiliations:** 1 Department of Clinical Sciences, Division of Internal Medicine, Danderyd Hospital, Karolinska Institutet, Stockholm, Sweden; 2 Division of Specialist Medical Care, Danderyd Hospital, Diagnostic Center, Stockholm, Sweden; 3 Department of Medical Cell Biology, SciLifeLab, Uppsala University, Uppsala, Sweden; 4 Department of Chemistry, University of Oxford, Oxford, UK; 5 Department of Medical Sciences, Clinical Chemistry and SciLifeLab Affinity Proteomics, Uppsala University, Uppsala, Sweden; 6 Division of Internal Medicine, Department of Clinical Sciences, Division of Orthopedics, Danderyd Hospital, Karolinska Institutet, Stockholm, Sweden; 7 Division of Hematology, Department of Medicine, UNC Blood Research Center, University of North Carolina at Chapel Hill, Chapel Hill, NC USA

**Keywords:** Neoplasms, Biomarkers, Histones, Plasma

## Abstract

Cancer detection is challenging, especially in patients with unspecific cancer symptoms. Biomarkers could identify patients at high risk of cancer. Prior studies indicate that neutrophil extracellular traps (NETs) are associated with cancer, but also with autoimmune and infectious diseases. The objective of this prospective study was to investigate markers associated with NET formation (nucleosomal citrullinated histone 3 [H3Cit-DNA], cell free DNA [cfDNA] and neutrophil elastase [NE]), and c-reactive protein (CRP) in patients with unspecific cancer symptoms, such as fatigue, weight loss or radiological sign of malignancy without an apparent primary tumor, referred to the Diagnostic Center at Danderyd Hospital in Sweden. Blood samples were drawn on admission, before cancer diagnosis. Out of 475 patients, 160 (34%) were diagnosed with cancer, 56 (12%) with autoimmune disease, 32 (7%) with infectious disease, 71 (15%) with other diseases and 156 (33%) received no diagnosis. H3Cit-DNA, cfDNA, NE and CRP were significantly higher in patients with cancer compared to patients without cancer (*p* < 0.0001, *p* < 0.0001, *p* = 0.004, and *p* = 0.0002 respectively). H3Cit-DNA, but not cfDNA, NE or CRP, was significantly elevated in patients with cancer compared to patients with autoimmune disease (*p* = 0.0001). H3Cit-DNA, cfDNA, NE or CRP did not differ between cancer and infectious disease. In conclusion, H3Cit-DNA is elevated in patients diagnosed with cancer compared to non-cancer patients with the same symptomatology. Further studies should evaluate if H3Cit-DNA could aid in selecting patients that would benefit the most from a rapid cancer diagnostic work-up.

**Graphical Abstract:**

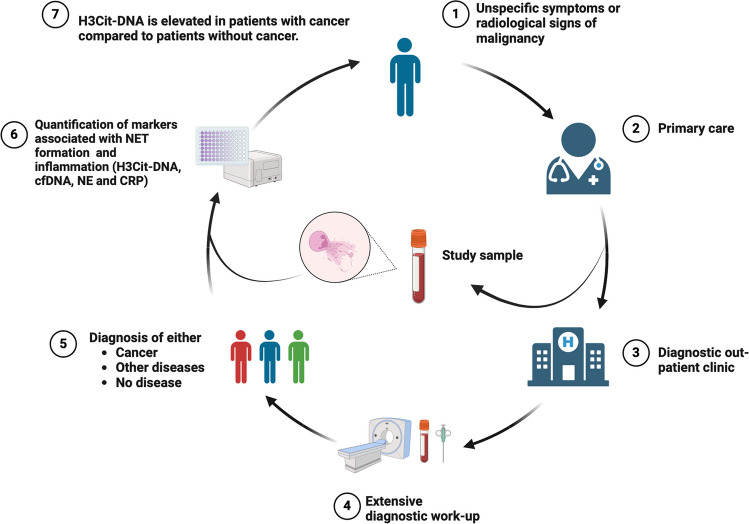

**Supplementary Information:**

The online version contains supplementary material available at 10.1007/s10753-024-02085-4.

## Introduction

Early cancer diagnosis and treatment are crucial in order to reduce cancer-related mortality and morbidity [1]. Unfortunately, almost half of all cancers are diagnosed when they have already metastasized [2]. Cancer detection is especially challenging in patients presenting with non-specific symptoms and signs of cancer [3, 4] because they are not eligible for investigation within one of the organ-specific cancer diagnostic pathways. This patient group may furthermore have an underlying non-malignant inflammation or infection giving rise to the unspecific symptoms [5, 6]. In addition, the well-established crosstalk between inflammatory processes and tumor development [7] hampers attempts to reliably distinguish between chronic inflammation and cancer. Several countries have therefore introduced standardized fast-track pathways with multidisciplinary diagnostic work-up for these patients [4, 6, 8]. The goal of these fast-track pathways is timely diagnosis and improved cancer survival. Extended and rapid cancer screening of all patients presenting with non-specific symptoms would, however, overload the healthcare system and burden patients with unnecessary and potentially harmful investigations. A low-cost and accessible blood test discriminating between patients with a high risk of cancer and those without cancer would therefore be of high clinical value.

Neutrophil extracellular traps (NETs) are web-like structures composed of DNA-strands coated with histones and granular proteins, such as neutrophil elastase (NE) and myeloperoxidase (MPO), released by activated neutrophils. Although initially described to entrap and disarm pathogens as a part of our innate immune system [9], NETs have been implicated in several non-infectious settings. Increasing evidence has demonstrated a role of NET formation in tumor progression and metastasis [10, 11] by facilitating metastatic spread, enhancing angiogenesis and aiding the escape of cancer cells from immune recognition by forming a barrier between cancer cells and the immune system [10, 12–16]. Peptidylarginine deaminase 4 (PAD4) is an enzyme primarily expressed in neutrophils and is involved in the release of NETs by catalyzing histone citrullination, which in turn initiates chromatin decondensation [17, 18]. Citrullinated histone H3 (H3Cit) and nucleosomal H3Cit (H3Cit-DNA) are thereby considered as markers of PAD4-dependent NET formation. Comprising part of the NET scaffold, cfDNA, NE and MPO have also been implied to reflect NET formation [19, 20]. However, NE reflects neutrophil activation independently of NET formation [20], and cfDNA has other origins than NETs, such as necrotic and apoptotic cells [21]. Although MPO-DNA complexes likely derive from chromatin-bound MPO, assays quantifying these complexes have recently been questioned due to low specificity [22] and H3Cit-DNA is currently considered the most specific biomarker for NET formation [22, 23].

Biomarkers associated with NET formation, including H3Cit, H3Cit-DNA, cfDNA and NE [24], have been found to be significantly elevated in peripheral blood from patients with cancer compared to healthy individuals [25–33], proposed as independent risk factors for occult cancer in patients with venous thromboembolism (VTE) [34] and as predictive indicators of cancer-associated VTE [35]. In addition, enhanced NET formation has been shown to correlate with increased tumor burden [27, 28] and poor prognosis in patients with cancer [31, 32, 36]. Biomarkers associated with NET formation have therefore been proposed as potential independent risk factors for occult cancer. However, most clinical studies investigating NETs in patients with cancer compare patients with a known cancer diagnosis to healthy individuals. Since several studies have implicated a role of NETs in autoimmune [37–39] and infectious diseases [9, 40], these findings may not be relevant in real life settings aiming to discriminate between patients with occult cancer and other non-malignant conditions.

The primary objective of this study was to compare the levels of circulating biomarkers of NET formation in patients with and without undiagnosed cancer in a cohort presenting with unspecific cancer symptoms. NET-associated biomarkers H3Cit-DNA, cfDNA and NE, along with the inflammatory marker C reactive protein (CRP), were quantified in blood samples obtained from 475 patients presenting with non-specific symptoms and signs of cancer prior to the multidisciplinary diagnostic work-up at the standardized fast-track diagnostic pathway at Danderyd Hospital, Stockholm, Sweden. Taking advantage of the high rate of patients diagnosed with a non-malignant autoimmune or infectious condition after the multidisciplinary diagnostic work-up, we further aimed to compare the levels of circulating markers of NET formation in patients diagnosed with cancer and those diagnosed with non-malignant infectious and autoimmune diseases.

## Method

### Study Population

**Fig. 1:**
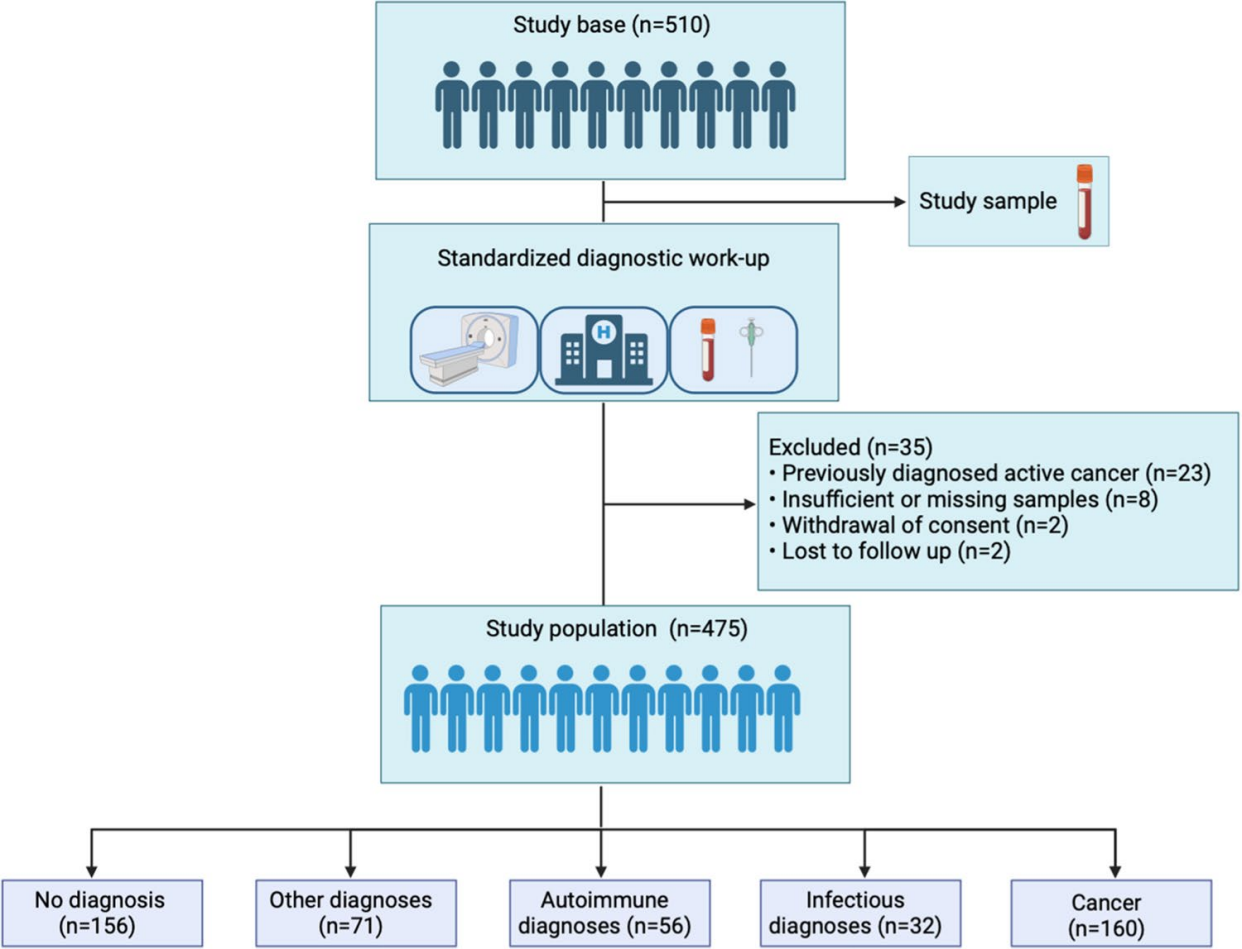
Flow chart of study participants.

Between March 2018 and October 2020, a total of 906 patients were admitted to the fast-track multidisciplinary diagnostic pathway at the Diagnostic center at Danderyd hospital. During this period, 510 patients were included in the study. Study enrollment was temporarily paused during holidays and at the onset of the covid-19 pandemic. Patients with known active cancer at inclusion (*n* = 23), patients who were lost to follow-up (*n* = 2), patients who withdrew consent (*n* = 2), and patients with insufficient plasma samples (*n* = 8) were excluded from further analysis (Fig. 1). Inclusion criteria were non-specific signs and symptoms of cancer with onset < 6 months prior to referral (*n* = 297) or radiological sign of malignancy without an apparent primary site (*n* = 178) and therefore not eligible for entrance into an organ-specific pathway. Non-specific signs and symptoms of cancer were defined as any of the following symptoms: general malaise, extreme fatigue, loss of appetite, unintentional weight loss, long lasting fever, unexplained pain, abnormal blood tests (such as anemia, elevated alkaline phosphatase, erythrocyte sedimentation rate or calcium levels), increased contacts to health system, increased use of medications or the impression that the patient is seriously ill, often referred to as ‘gut feeling’. All study patients underwent a standardized and extensive cancer diagnostic work up, including an expanded panel of biochemical analyses, diagnostic tissue biopsies and imaging such as computed tomography, magnetic resonance or ^18^F fluorodeoxyglucose positron emission tomography/computed tomography investigations. Demographic data, comorbidity, cancer diagnosis and other diagnoses were obtained from hospital records.

#### Outcome

Patients were followed for six months from study inclusion regarding new cancer diagnosis and other diagnoses. Diagnoses were obtained from individual medical records. Basal cell carcinoma diagnosis was not obtained from medical records as it does not share the metastatic features of other cancers.

### Laboratory Analyses

Venous blood samples were collected at enrollment at the first visit to the Diagnostic center (*i.e.* before cancer diagnostic work-up). Citrated plasma samples were centrifugated for 20 min at 2000 × g at room temperature immediately following sampling and were stored at -80 °C until further analysis. H3Cit-DNA was quantified using a recently validated in-house ELISA [23]. This method uses a monoclonal capture antibody (Cat# ab232939, Abcam) with a high specificity for citrullinated histones and low inter-lot variability [23], a dsDNA detection antibody (DNA POD, part of Cell Death Detection ELISA PLUS kit, Roche Cat#11774425001) and semi-synthetic nucleosome calibrators (H3R2,8,17Cit dNuc, Epicypher, Cat#16–1362) to overcome stability issues of histones in plasma as well as the high inter-lot variability of enzymatically modified histones [23]. CRP was analyzed using Cobas 6000 (Roche Diagnostics), NE was quantified using the PMN Elastase Human ELISA Kit (Cat# ab119553, Abcam) and cfDNA was quantified using the Quant-iT Picogreen assay (Cat# P7589, Invitrogen) according to manufacturer’s instructions.

### Statistical analyses

Statistical analyses were performed in STATA version 17.0 and GraphPad Prism version 9. Parametric data is presented as mean (standard deviation [SD]) and non-parametric data as median (interquartile range [IQR]). Mann Whitney U test was used for comparisons between blood biomarkers as these were not normally distributed. Uni- and multivariable logistic regression were performed using the level of circulating biomarkers as Log2-transformed continuous variables. In multivariable analysis, adjustment was made for sex, age, BMI, current smoking, autoimmune disease, COPD, diabetes, arterial disease, and previous cancer. All tests were 2-sided and p < 0.05 was considered statistically significant.

### Data Sharing Statement

The datasets generated during the current study are not publicly available due to privacy concerns and limitations from the ethical review board but are available from the corresponding author on reasonable request.

## Results

### Patient Characteristics

The study population comprised 475 patients (55% female; mean age 68.0 (SD 14.0) years) (Table 1). A total of 160 patients (34% of total; 53% female; mean age 71.5 [SD 13.0] years) were diagnosed with cancer during the 6-month follow-up period for cancer. The most common cancer diagnoses were hematologic malignancies (28%), pancreas, gall bladder and bile duct adenocarcinomas (11%) and lung adenocarcinomas (8%) (Table 2). Metastatic disease was found in 101 out of 115 patients with solid tumors (86%). Primary tumor origin was not confirmed in 13 patients, either due to inconclusive histopathology (*n* = 7), hard-to-reach tissue (*n* = 1) or patient withdrawal from further investigations due to poor prognosis (*n* = 5). Levels of H3Cit-DNA, cfDNA, NE and CRP for different cancer types, metastatic and non-metastatic disease, solid tumors and hematological malignancies are presented in Supplementary Tables S1-S3. Out of the 315 patients who did not receive a cancer diagnosis during the follow-up period (66% of total; 55% female; mean age 66.2 (SD 14.2) years), 159 patients were diagnosed with a non-malignant disease (Supplementary Table S4) and 156 patients did not receive a diagnosis during the follow-up period. Out of the 159 patients that were diagnosed with a non-malignant disease, 56 (12%) patients received an autoimmune diagnosis, 32 (7%) patients were diagnosed with an infectious disease, and 71 (15%) patients were diagnosed with other diseases. Further division of non-malignant diagnoses are presented in Supplementary Table S4. Baseline data of patients grouped according to outcome is presented in Table 1 and Supplementary Table S5.

**Table 1:** Baseline Characteristics of Patients According to outcome during Follow-up. Results are Presented as Number (%) or Median (IQR)

	All patients (*n* = 475)	Cancer diagnoses (*n* = 160)	No cancer diagnosis (*n* = 315)	Infectious diagnoses (*n* = 32)	Autoimmune diagnoses (*n* = 56)_a_
Female sex	260 (55)	85 (53)	175 (56)	10 (31)	31 (55)
Age	71 (60–78)	74 (66–81)	70 (57–76)	72 (60–80)	70 (61–76)
BMI	25 (22–28)	25 (22–28)	25 (22–28)	25 (23–29)	25 (22–28)
Current smoking	57 (12)	19 (12)	38 (12)	2 (6)	7 (13)
Autoimmune disease_a_	80 (17)	17 (11)	63 (20)	4 (13)	9 (16)
COPD	50 (11)	14 (9)	36 (11)	7 (22)	5 (9)
DM	74 (16)	23 (14)	51 (16)	9 (29)	9 (16)
Arterial disease	82 (17)	30 (19)	52 (16)	7 (22)	9 (16)
Previous cancer	81 (17)	29 (18)	52 (16)	7 (22)	8 (14)
CRP (mg/L)_b_	5 (1–30)	10 (1.8–43)	4 (1–15)	10 (4–87)	20 (3–82)
H3Cit-DNA (ng/mL)	106 (74–161)	128 (89–220)	96 (69–140)	114 (84–157)	92 (68–132)
cfDNA (ng/mL)	436 (389–488)	452 (415–521)	427 (378–471)	459 (431–493)	441 (390–507)
NE (ng/mL)	24 (17–36)	29 (19–39)	23 (17–33)	31 (17–47)	31 (22–41)

_a_Hypothyroidism not included. _b_Available for 464 patients. IQR, inter quartile range; BMI, body mass index; COPD, chronic obstructive pulmonary disease; DM, diabetes type 1 or type 2; H3Cit-DNA, nucleosomal citrullinated histone H3; cfDNA, cell free DNA; NE, neutrophil elastase

**Table 2:** Cancer Types Diagnosed during Follow-up

Cancer site_a_	Cancer during follow up (n = 160_b_)	Metastases at diagnosis (*n* = 101_b_)
Carcinoma, No (%)	100 (63)	91 (91)
Germinal, No (%)	2 (1.3)	2 (100)
Urothelial, No (%)	4 (2.5)	3 (75)
Neuroendocrine tumor, No (%)	12 (7.5)	9 (75)
Adenocarcinoma, No (%)	81 (50.6)	71 (89)
Breast, No(%)	9 (5.6)	8 (89)
HCC, No(%)	6 (3.8)	4 (67)
Kidney, No (%)	7 (4.4)	5 (83)
Colorectal No (%)	5 (3.1)	5 (100)
Prostate, No (%)	4 (2.5)	2 (50)
Lung, No (%)	13 (8.1)	11 (85)
Gastric, No (%)	3 (1.9)	3 (100)
Thyroid, No (%)	4 (2.5)	3 (75)
Ovaries and tubar, No (%)	8 (5)	8 (100)
Pancreas, gall bladder and bile duct, No (%)	17 (11)	17 (100)
Parotid, No (%)	1 (0.63)	1 (100)
Appendix, No (%)	2 (1.3)	2 (100)
Pseudomyxoma, No (%)	1 (0.63)	1 (100)
Adenocarcinoma with unknown primary, No (%)	3 (1.9)	3 (100)
Mesothelioma, No (%)	3 (1.9)	3 (1.9)
Carcinoma with unknown primary	3 (1.9)	3 (100)
Sarcoma, No (%)	4 (2.5)	3 (75)
GIST, No (%)	1 (0.63)	0 (0)
Other sarcoma, No (%)	3 (1.9)	3 (75)
Myeloma, No (%)	16 (10)	NA
Lymphoma, No (%)	24 (15)	NA
B-cell-lymphoma, No (%)	7 (4.4)	NA
T-cell lymphoma, No (%)	2 (1.3)	NA
Follicular lymphoma, No (%)	4 (2.5)	NA
Hodgkins lymphoma, No (%)	5 (3.1)	NA
Mantle cell lymphoma, No (%)	2 (1.3)	NA
Waldenstrom macroglobulinemia, No (%)	1 (0.63)	NA
Chronic lymphocytic leukemia, No (%)	3 (1.9)	NA
MDS, No (%)	4 (2.5)	NA
Myeloproliferative neoplasm, No (%)	1 (0.63)	NA
Melanoma	0 (0)	0 (0)
Glioblastoma	1 (0.63)	1 (100)
Cancer with unknown primary	7 (4.4)	7 (100)

GIST, gastrointestinal stromal tumor; MDS, myelodysplastic syndrome; NA; Not applicable

_a_Categorized according to Swedish national cancer care program for cancers without known primary [41, 42]

_b_The total number exceeds 160 and 101 respectively, as some patients had more than one primary tumor

### Circulating markers of NET formation in patients presenting with non-specific symptoms and signs of cancer with and without a cancer diagnosis following the multidisciplinary diagnostic work-up

**Fig. 2:**
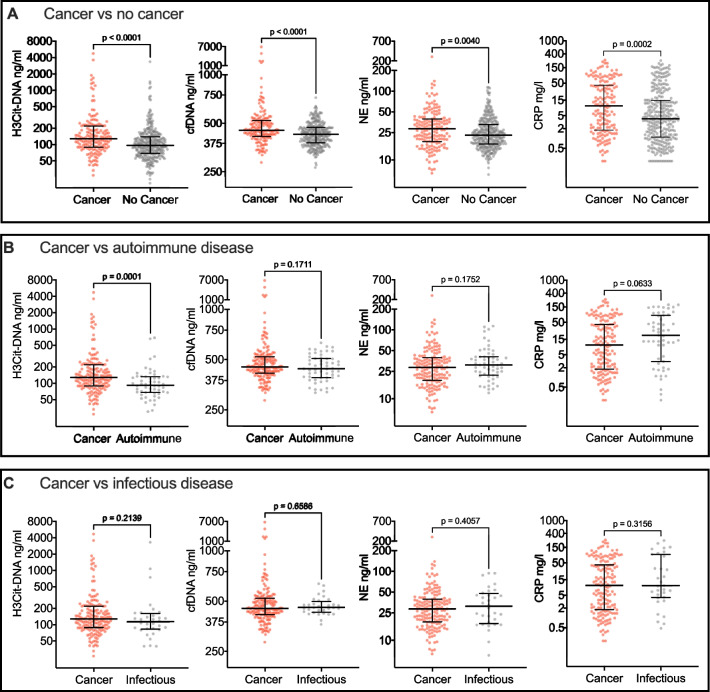
Levels of H3Cit-DNA, cfDNA, NE and CRP in patients with cancer (*n* = 160) and no cancer (*n* = 315) during diagnostic follow-up, autoimmune diagnoses (*n* = 56) and infectious diagnoses (*n* = 32). *H3Cit-DNA*, Nucleosomal Citrullinated Histone H3; cfDNA, cell free DNA; NE, neutrophil elastase; *CRP,* C-reactive protein. Lines represent median and IQR. Y-axes are plotted on a log2 scale. Groups were compared with the Mann–Whitney U test. Autoimmune diagnoses that occurred more than once: polymyalgia rheumatica (*n* = 10), giant cell vasculitis (*n* = 11), sarcoidosis (*n* = 7), rheumatoid arthritis (*n* = 7), primary biliary cholangitis (*n* = 3), autoimmune hepatitis (*n* = 2), systemic lupus erythematosus (*n* = 2), psoriasis arthritis (*n* = 2). Other autoimmune diagnoses occurred only once. Infectious diagnoses were bacterial (*n* = 24), viral (*n* = 6) or fungal (*n* = 2).

Plasma levels of H3Cit-DNA, cfDNA, and NE were significantly higher in the 160 patients diagnosed with cancer compared to the 315 patients presenting with the same symptoms and not diagnosed with cancer (median 128 [89–220] ng/ml vs 96 [69–140] ng/ml for H3Cit-DNA, *p* < 0.0001, 452 [415–521] ng/ml vs 427 [378–471] ng/ml for cfDNA, *p* < 0.0001 and 29 [19–39] ng/ml vs 23 [17–33] ng/ml for NE, *p* = 0.004 (Fig. 2A). Similar differences were observed for CRP with a median of 10 (1.8–43) mg/l in patients diagnosed with cancer and a median of 4 (1–15) mg/l in patients not diagnosed with cancer, *p* = 0.0002 (Fig. 2A). To control for confounding variables, we also performed uni- and multivariable logistic regression adjusting for sex, age, BMI, current smoking, autoimmune disease, COPD, diabetes, arterial disease and previous cancer, with similar results and significant differences between patients diagnosed with cancer compared to patients presenting with the same symptoms and not diagnosed with cancer in all biomarkers (Supplementary Table S6). Levels of H3Cit-DNA and NE were similar in patients with metastatic and non-metastatic disease, but levels of cfDNA and CRP were higher in patients with metastatic disease (Supplementary Table S2). However, there were only a small number of patients with non-metastatic solid tumors (12% of patients with solid tumors), and as these patients had systemic symptoms, our results may not be comparable to other studies including patients without systemic symptoms. We did not compare levels of biomarkers between different cancer types, as there were few in each subgroup and stages varied greatly between cancer types.

### Circulating markers of NET formation in patients presenting with non-specific symptoms and signs of cancer receiving a cancer and non-malignant infectious or autoimmune diagnosis following the multidisciplinary diagnostic work-up

We next compared the levels of circulating markers of NET formation in patients diagnosed with cancer (*n* = 160) and non-malignant infectious (*n* = 32) or autoimmune (*n* = 56) diagnoses following the multidisciplinary diagnostic work-up. Interestingly, H3Cit-DNA, but not cfDNA and NE, was significantly elevated in patients diagnosed with cancer compared to patients diagnosed with autoimmune disease (median 128 [89–220] ng/ml vs 92 [68–132] ng/ml for H3Cit-DNA, *p* = 0.0001, 452 [415–521] ng/ml vs 441 ng/ml [390–507] for cfDNA, *p* = 0.17 and 29 ng/ml [19–39] vs 31 ng/ml [22–41] for NE, *p* = 0.18). In contrast, CRP trended towards higher levels in patients diagnosed with autoimmune disease compared to patients diagnosed with cancer (median 20 [3–82] mg/l vs 10 [1.8–43] ng/ml, *p* = 0.06), likely reflecting more inflammation in patients with autoimmune disease (Fig. 2B). Notably, 17 of the 160 patients diagnosed with cancer had a confirmed autoimmune disease prior to inclusion, and exclusion of these patients did not reduce the observed differences in H3Cit-DNA between patients with cancer and autoimmune diseases (median 135 [90–222] ng/ml vs 92 [68–132] ng/ml, *p* < 0.0001) or CRP (median 9.9 [2.1–44.3] mg/l vs 22.5 [3.1–82.0] mg/l, *p* = 0.06). No significant differences were observed in the levels of H3Cit-DNA, cfDNA, NE or CRP when comparing patients receiving a cancer diagnosis to patients receiving a non-malignant infectious diagnosis (p = 0.21, 0.66, 0.41 and 0.32 respectively) (Fig. 2C). Uni- and multivariable logistic regression adjusting for sex and age (for cancer vs infectious disease) and sex, age, current smoking and previous cancer (for cancer vs autoimmune disease) with similar results (Supplementary Table S6). Taken together, only H3Cit-DNA show potential to discriminate between patients diagnosed with cancer and patients not diagnosed with cancer as well as patients diagnosed with cancer and autoimmune disease. No markers associated with NET formation were able to discriminate between patients diagnosed with cancer and patients receiving a diagnosis of an infectious disease.

## Discussion

Several studies have demonstrated elevated levels of markers of NET formation in patients with cancer compared to healthy individuals. Here, we report that the NET associated markers H3Cit-DNA, cfDNA and NE are elevated in a well-characterized cohort of patients diagnosed with cancer compared to patients presenting with the same symptoms diagnosed with non-malignant conditions. Importantly, only the NET-specific biomarker H3Cit-DNA, but not cfDNA and NE, was significantly higher in patients diagnosed with cancer compared to patients referred to the diagnostic outpatient clinic due to unspecific cancer symptoms but diagnosed with autoimmune conditions, a patient population extremely difficult to distinguish from patients with occult cancer. In contrast, the levels of H3Cit-DNA, cfDNA, NE and CRP did not differ between patients diagnosed with cancer and non-malignant infectious conditions, suggesting that H3Cit-DNA may reflect an underlying cancer in non-infectious settings.

NETs are released through multiple and still poorly understood mechanisms. PAD4 triggers chromatin decondensation and NET formation [17, 18] and H3Cit is therefore considered a marker of PAD4-dependent NET formation [24]. Mutation of PAD4 (PAD4C645S) has been demonstrated to abolish NET formation in the human cancer cell line HL-60 after calcium ionophore stimulation [13], and the PAD4 inhibitor GSK484 has been shown to reduce NET formation in both human and mouse neutrophils [17]. However, PMA-induced NET formation has been shown without histone H3 citrullination, and the pan-PAD inhibitors Cl-amidine and BB-Cl-amidine did not impede *in vitro* NET formation induced by a variety of agents. These findings indicate both PAD4-dependent and independent pathways of NET formation, emphasizing that the absence of citrullinated histones may not rule out NET formation.

H3Cit-DNA, cfDNA, NE and CRP were all significantly elevated in patients diagnosed with cancer compared to patients not diagnosed with cancer. When comparing patients diagnosed with cancer to patients diagnosed with autoimmune disease, only H3Cit-DNA was able to discriminate between the two groups. None of the investigated markers were able to discriminate between patients diagnosed with cancer and patients diagnosed with infectious disease. Our findings of elevated levels of H3Cit-DNA in patients with cancer compared to patients receiving a non-malignant autoimmune diagnosis but comparable levels in cancer and non-infectious diagnoses may suggest similar pathways of NET induction in cancer and infection but distinct pathways of NET formation in cancer and autoimmune settings. A partial cancer cell origin of H3Cit-DNA in cancer patients can furthermore not be ruled out, as various tumor cells express PAD4, and these cells have been demonstrated to extrude chromatin containing citrullinated histones [43].

One third of the patients in our cohort received a cancer diagnosis during the diagnostic work-up. Previous studies, mainly from Sweden and Denmark where standardized pathways for patients with non-specific symptoms and signs of cancer are well established, have reported that 11–35% [4] of referred patients are diagnosed with cancer. The difference in cancer rates is likely due to different national and international referral criteria. This study included both patients who were referred due to non-specific signs and symptoms of cancer and patients with radiological findings suggestive of metastases without known primary tumor, which may explain the high cancer rate. The most common cancer types in our cohort were hematologic malignancies, followed by pancreas, gall bladder and bile duct adenocarcinomas and lung adenocarcinomas. These findings are in line with previous studies, apart from a lower rate of colorectal cancer cases [4]. Patients with hematological malignancies often present with symptoms that are not organ-specific, resulting in the high rate of hematological malignancies in this cohort compared to the general cancer panorama.

This study is strengthened by the prospective cohort design with sample collection prior to cancer diagnostic work-up, diagnosis and treatment. Study participants are well characterized after the extensive and multidisciplinary diagnostic work-up, reducing the risk of misclassifications. Only a few patients were excluded due to missing data or plasma samples. Several studies have measured H3Cit in plasma samples showing its elevation and prognostic value in various pathologic conditions [37–40]. However, we recently reported a high enzyme-dependent lot variability for enzymatically citrullinated H3 proteins as well as instability of these histones in plasma [23]. We furthermore demonstrated a poor specificity in the majority of commercially available antibodies targeting intrapeptidyl citrulline [23] which may explain the high variability between studies in the reported levels of circulating H3Cit in human plasma. H3Cit-DNA in the samples obtained for this study were therefore quantified with our recently thoroughly validated assay [23] utilizing highly specific monoclonal antibodies and semi-synthetic nucleosomes containing citrulline in place of arginine at histone H3, arginine residues 2, 8, and 17 (H3R2,8,17Cit) as calibration standard, which reliably quantifies nucleosomal H3Cit in human plasma. Although we therefore believe that we have overcome some of the challenges in quantifying H3Cit in plasma, quantifying citrullinated histones is challenging. We furthermore did not quantify other markers associated with NET formation such as myeloperoxidase (MPO) [44] and MPO-DNA [26]. Moreover, our findings cannot be extrapolated to patients with localized malignant diseases since the majority of study participants diagnosed with cancer were diagnosed with metastatic disease or hematological malignancy. The sample size was furthermore not large enough to allow comparisons between cancer types, or between patients with cancer and patients with non-autoimmune inflammatory disease, which further limits the generalizability of the results. However, the concept of fast-track multidisciplinary pathways to reduce the time to diagnosis of cancer in patients with unspecific symptoms is growing and are now available in several countries, and our results are generalizable to this patient group.

Taken together, our findings support prior evidence on the role of NET formation in cancer biology. Additionally, our study expands on these findings by demonstrating clear elevations of circulating H3Cit-DNA in patients diagnosed with cancer compared to patients diagnosed with non-malignant autoimmune disorders, despite having the same symptoms. Although the origin of circulating H3Cit-DNA lies beyond the scope of this study, our findings suggest distinct pathways of NET formation in malignant and autoimmune settings. Further studies are needed to investigate if H3Cit-DNA, alone or in combination with other risk markers, could aid in selecting patients that would benefit the most from a rapid cancer diagnostic work-up.

## Supplementary Information

Below is the link to the electronic supplementary material.

Supplementary file1 (PDF 131 KB)

Supplementary file2 (PDF 365 KB)

## Data Availability

The datasets generated during the current study are not publicly available due to privacy concerns and limitations from the ethical review board but are available from the corresponding author on reasonable request.
