# Supplementary material for: Plasma H3Cit-DNA Discriminates Between Cancer and Inflammation in a Cohort of Patients with Unspecific Cancer Symptoms
Source: Inflammation. 2024 Jun 28;48(2):760–9. doi: 10.1007/s10753-024-02085-4 (PMC12053196; doi:10.1007/s10753-024-02085-4)
Supplement: Supplementary file 2 — Supplementary file2 (PDF 365 KB) [file 10753_2024_2085_MOESM2_ESM.pdf]

SUPPLEMENTAL APPENDIX

Plasma H3Cit-DNA discriminates cancer from inflammation in a cohort presenting with unspecific symptoms

Fredrika Wannberg[1], Viktoria Hjalmar[1,3], Henry Ng[1, 2], Caroline Johansson[3], Fay Probert[4], Mia Philipson[2], Mikael Åberg[5], Max Gordon[6], Nigel Mackman[7], Axel Rosell*[1], Charlotte Thålin*[1.]

1. Department of Clinical Sciences, Danderyd Hospital, Division of Internal Medicine, Karolinska Institutet, Stockholm, Sweden
2. Department of Medical Cell Biology, SciLifeLab, Uppsala University, Uppsala Sweden
3. Division of Specialist Medical Care, Diagnostic center, Danderyd Hospital, Stockholm, Sweden
4. Department of Chemistry, University of Oxford, Oxford, United Kingdom
5. Department of Medical Sciences, Clinical Chemistry and SciLifeLab Affinity Proteomics, Uppsala University, Uppsala, Sweden
6. Department of Clinical Sciences, Danderyd Hospital, Division of Orthopedics, Karolinska Institutet, Stockholm, Sweden
7. UNC Blood Research Center, Division of Hematology, Department of Medicine, University of North Carolina at Chapel Hill, Chapel Hill, North Carolina, USA

*Equal contribution.

Corresponding author:
Charlotte Thålin,
Division of Internal Medicine, Department of Clinical Sciences, Danderyd Hospital,
Karolinska Institutet, Stockholm, Sweden
charlotte.thalin@ki.se

Table S1. Levels of H3Cit-DNA, cfDNA, NE and CRP in patients with different cancer types (results presented as median with IQR).

	Adenocarcinoma (n=80)	Neuroendocrine (n=12)	Lymphoma (n=24)	Myeloma (n=16)	Non-adenocarcinoma solid tumors (n=27)
H3Cit-DNA (ng/mL)	142 (95–230)	114 (80.9–155)	124 (87.5–218)	105 (74.2–140)	133 (89–176)
cfDNA (ng/mL)	475 (422–533)	448 (408–489)	441 (397–522)	450 (411–522)	444 (405–475)
NE (ng/mL)	30 (20–41)	20 (14–28)	25 (18–42)	21 (17–29)	29 (15–38)
CRP (mg/L)	16.0 (2.6–56.0)	2.3 (0.8–9.9)	4.3 (0.9–59.0)	3.9 (1.1–8.8)	5.4 (1.3–41)

H3Cit-DNA, Nucleosomal Citrullinated Histone H3; cfDNA, cell free DNA; NE, neutrophil elastase; *CRP,* C-reactive protein

Table S2. Levels of H3Cit-DNA, cfDNA, NE and CRP in patients with metastatic solid tumor and non-metastatic solid tumor (results presented as median with IQR)

	Metastatic solid tumor (n=101)	Non-metastatic solid tumor (n=14)	P-value for difference
H3Cit-DNA (ng/mL)	137 (95.5–222)	138 (90.6–227)	0.63
cfDNA (ng/mL)	469 (426–535)	427 (376–478)	0.02
NE (ng/mL)	31 (20–43)	30 (20–37)	0.47
CRP (mg/L)	17.0 (2.85–64.0)	3.30 (1.00–11.0)	0.006

H3Cit-DNA, Nucleosomal Citrullinated Histone H3; cfDNA, cell free DNA; NE, neutrophil elastase; *CRP,* C-reactive protein. Groups were compared with the Mann Whitney U test.

Table S3. Levels of H3Cit-DNA, cfDNA, NE and CRP in patients with solid tumor vs hematological malignancies (results presented as median with IQR)

	Solid tumors (n=115)	Hematological malignancies (n=45)	P-value for difference
H3Cit-DNA (ng/mL)	137 (91.2–227)	114 (83.1–165)	0.077
cfDNA (ng/mL)	457 (422–523)	467 (403–505)	0.19
NE (ng/mL)	30 (20–42)	22 (16–34)	0.062
CRP (mg/L)	13.5 (2.60–56.0)	3.30 (1.00–35.0)	0.0062

H3Cit-DNA, Nucleosomal Citrullinated Histone H3; cfDNA, cell free DNA; NE, neutrophil elastase; *CRP,* C-reactive protein. Groups were compared with the Mann Whitney U test.

Table S4. Non-malignant diagnoses among patients without cancer during follow-up (n=159).

Diagnosis	Number of patients[a]
Infections	32
Bacterial	24
Viral	6
Fungal	2
Parodontitis	10
Autoimmune disease	56
Polymyalgia rheumatica	10
Giant cell arteritis/vasculitis	11
Small vessel vasculitis	3
Rheumatoid arthritis/polyarthritis/monoarthritis	7
Sarcoidosis	7
Autoimmune thyroid gland diseases	3
Primary biliary cholangitis	3
Autoimmune hepatitis	2
Microscopic polyangiitis	2
Psoriatic arthritis	2
Systemic lupus erythematosus	2
Alcohol dependency	8
Gastrointestinal inflammation (Barrets, gastritis, duodenitis and diverticulitis)	8
Osteoporosis with or without pathological fractures	6
Pericardial effusion	6
Heart failure	5
Liver cirrhosis	4
Venous thromboembolism	4
Kidney failure	3
Ureter/kidney stone	3
Alcoholic liver disease (no cirrhosis)	2
Iron deficiency due to gastrointestinal bleeding	2

List of diagnoses that occurred once. Autoimmune diseases: Autoimmune pancreatitis, celiac disease, Grave's disease, myositis, morphea, polyarteritis nodosa, psoriasis, Paget's disease of bone, pelvospondylitis, retroperitoneal fibrosis, systemic inflammatory disease unspecified[b]. Other diagnoses: aseptic lymphocyte-dominant vasculitis-associated lesion, bursitis, chronic osteomyelitis, endometriosis, femoral head necrosis, foreign body granuloma in colon, gout, hidradenitis suppurativa, inflammation in thigh of unknown cause, Langerhans' cell histiocytosis, liver cysts with inferior vena cava compression, lumbar disc herniation with reactive lymph nodes, meningioma, necrotizing granuloma of the liver of unknown cause, pancreatic insufficiency, pleural effusion, polycystic ovaries, primary hyperparathyroidism, rectal adenoma with high grade dysplasia, small bowel ischemia, spinal hemangioma, substance abuse (anabolic steroids), Wernicke encephalopathy.

[a]The total number of diagnoses (162) exceeds 159 as some patients received more than one diagnosis.

[b]High erythrocyte sedimentation rate (ESR), was treated with Methotrexate

Table S5. Baseline characteristics of patients according to outcome during follow-up. Results are presented as number (%) or median (IQR).

	**Cancer diagnoses (n=160)**	**No cancer diagnosis (n=315)**	**Infectious diagnoses (n=32)**	**Autoimmune diagnoses (n=56)[a]**	**Other diagnoses (n=71)**	**No abnormality detected (n=156)**
Female sex	85 (53)	175 (56)	10 (31)	31 (55)	36 (51)	98 (62)
Age	74 (66–81)	70 (57–76)	72 (60–80)	70 (61–76)	70 (58–76)	69 (55–77)
BMI	25 (22–28)	25 (22–28)	25 (23–29)	25 (22–28)	26 (22–31)	24 (22–27)
Current smoking	19 (12)	38 (12)	2 (6)	7 (13)	9 (13)	20 (13)
Autoimmune disease[a]	17 (11)	63 (20)	4 (13)	9 (16)	16 (23)	34 (22)
COPD	14 (9)	36 (11)	7 (22)	5 (9)	10 (14)	14 (9)
DM	23 (14)	51 (16)	9 (29)	9 (16)	13 (18)	20 (13)
Arterial disease	30 (19)	52 (16)	7 (22)	9 (16)	15 (	21 (13)
Previous cancer	29 (18)	52 (16)	7 (22)	8 (14)	11 (15)	26 (17)
CRP (mg/L)[b]	10 (1.8–43)	4 (1–15)	10 (4–87)	20 (3–82)	6 (2–25)	2 (1–5)
H3Cit-DNA (ng/mL)	128 (89–220)	96 (69–140)	114 (84–157)	92 (68–132)	110 (79–167)	89 (65–125)
cfDNA (ng/mL)	452 (415-521)	427 (378–471)	459 (431–493)	441 (390–507)	435 (394–499)	402 (363–452)
NE (ng/mL)	29 (19–39)	23 (17–33)	31 (17–47)	31 (22–41)	25 (18–33)	20 (16–26)

[a]Hypothyroidism not included. IQR, inter quartile range; BMI, body mass index; COPD, chronic obstructive pulmonary disease; DM, diabetes type 1 or type 2; H3Cit-DNA, nucleosomal citrullinated histone H3; cfDNA, cell free DNA; NE, neutrophil elastase

Supplemental Table S6. Association between H3Cit-DNA, cfDNA, NE and CRP and cancer during follow-up in uni- multivariable logistic regression models.

**Analysis**	**Variable**	**OR**	95% CI	*p*-value
Univariable Cancer vs no cancer	H3Cit-DNA (per 2-fold increase)	1.55	1.29–1.86	$3.8 \cdot 10^{-6}$
	cfDNA (per 2-fold increase)	6.64	3.13–14.1	$7.9 \cdot 10^{-8}$
	NE (per 2-fold increase)	1.41	1.11–1.79	0.0048
	CRP (per 2-fold increase)	1.15	1.07–1.24	$1.9 \cdot 10^{-4}$
Multivariable[a] Cancer vs no cancer	H3Cit-DNA (per 2-fold increase)	1.50	1.24–1.83	$3.5 \cdot 10^{-5}$
	cfDNA (per 2-fold increase)	6.03	2.75–13.2	$7.1 \cdot 10^{-6}$
	NE (per 2-fold increase)	1.35	1.05–1.73	0.021
	CRP (per 2-fold increase)	1.12	1.04–1.21	0.0038
Univariable Cancer vs infectious disease	H3Cit-DNA (per 2-fold increase)	1.14	0.82–1.60	0.428
	cfDNA (per 2-fold increase)	1.35	0.51–3.54	0.548
	NE (per 2-fold increase)	0.88	0.58–1.34	0.560
	CRP (per 2-fold increase)	0.93	0.80–1.07	0.320
Multivariable[b] Cancer vs infectious disease	H3Cit-DNA (per 2-fold increase)	1.12	0.80–1.55	0.518
	cfDNA (per 2-fold increase)	1.20	0.45–3.20	0.716
	NE (per 2-fold increase)	0.85	0.55–1.33	0.482
	CRP (per 2-fold increase)	0.92	0.79–1.06	0.243
Univariable Cancer vs autoimmune disease	H3Cit-DNA (per 2-fold increase)	1.84	1.28–2.65	0.0011
	cfDNA (per 2-fold increase)	2.58	0.94–7.13	0.067
	NE (per 2-fold increase)	0.79	0.56–1.12	0.185
	CRP (per 2-fold increase)	0.90	0.80–1.01	0.084
Multivariable[c] Cancer vs autoimmune disease	H3Cit-DNA (per 2-fold increase)	1.83	1.26–2.65	0.0013
	cfDNA (per 2-fold increase)	2.38	0.88–6.47	0.088
	NE (per 2-fold increase)	0.73	0.50–1.05	0.090
	CRP (per 2-fold increase)	0.88	0.78–0.99	0.034

OR, odds ratio; CI, confidence interval; H3Cit-DNA, Nucleosomal Citrullinated Histone H3; cfDNA, cell-free DNA; NE, neutrophil elastase; CRP, C-reactive protein. [a]Adjusted for sex, age, body mass index, current smoking, autoimmune disease, chronic obstructive pulmonary disease, diabetes, arterial disease and previous cancer. [b]Adjusted for sex and age. [c]Adjusted for sex, age, current smoking and previous cancer.

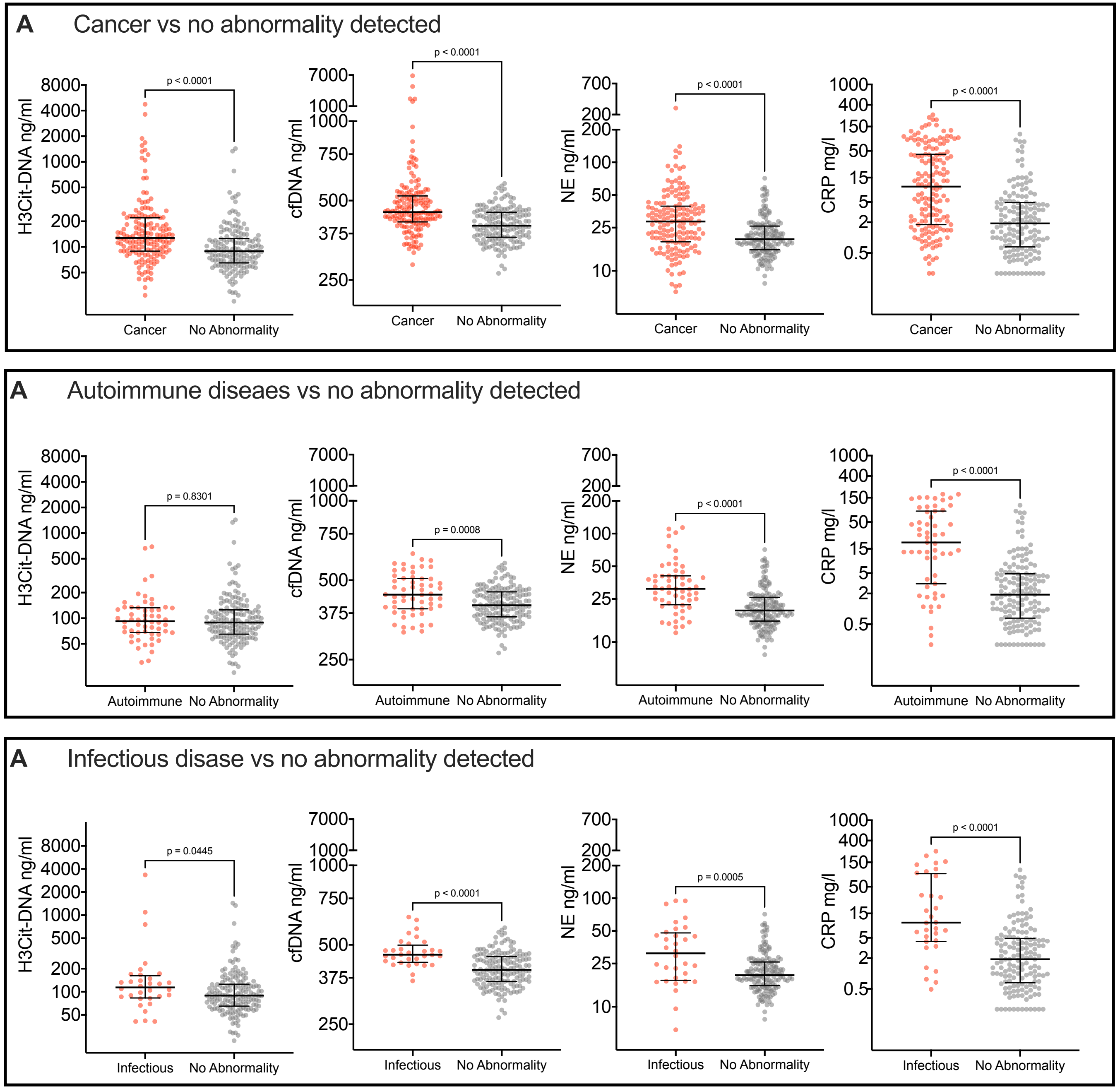
A
Cancer vs no abnormality detected
H3Cit-DNA ng/ml
cfDNA ng/ml
NE ng/ml
CRP mg/l
p < 0.0001
Cancer
No Abnormality
A
Autoimmune diseaes vs no abnormality detected
p = 0.8301
p = 0.0008
p < 0.0001
Autoimmune
No Abnormality
A
Infectious disase vs no abnormality detected
p = 0.0445
p < 0.0001
p = 0.0005
Infectious
No Abnormality

Figure S1. Levels of H3Cit-DNA, cfDNA, NE and CRP in patients with cancer (n=160) during diagnostic follow-up, autoimmune diagnoses (n=56), infectious diagnoses (n=32) and in patients with no abnormality detected (n=156). *H3Cit-DNA*, Nucleosomal Citrullinated Histone H3; cfDNA, cell free DNA; NE, neutrophil elastase; *CRP,* C-reactive protein. Lines represent median and IQR. Y-axes are plotted on a log2 scale. Groups were compared with the Mann-Whitney U test. Autoimmune diagnoses that occurred more than once: polymyalgia rheumatica (n=10), giant cell vasculitis (n=11), sarcoidosis (n=7), rheumatoid arthritis (n=7), primary biliary cholangitis (n=3), autoimmune hepatitis (n=2), systemic lupus erythematosus (n=2), psoriasis arthritis (n=2). Other autoimmune diagnoses occurred only once. Infectious diagnoses were bacterial (n=24), viral (n=6) or fungal (n=2).
